# Exogenous Brassinosteroid Enhances Zinc tolerance by activating the Phenylpropanoid Biosynthesis pathway in Citrullus lanatus L

**DOI:** 10.1080/15592324.2023.2186640

**Published:** 2023-04-21

**Authors:** Xuefang Liu, Quanwen Zhu, Wentao Liu, Jun Zhang

**Affiliations:** a College of Resources and Environmental Engineering, Yangzhou Polytechnic College, Yangzhou, China; b Jiangsu Safety & Environment Technology and Equipment for Planting and Breeding Industry Engineering Research Center, Yangzhou, China

**Keywords:** 24-epibrassinolide, Heavy metal, Oxidative damage, Phenylpropanoid biosynthesis, RNA-seq, Watermelon, Zinc stress

## Abstract

Zinc (Zn) is an important element in plants, but over-accumulation of Zn is harmful. The phytohormone brassinosteroids (BRs) play a key role in regulating plant growth, development, and response to stress. However, the role of BRs in watermelon (*Citrullus lanatus* L.) under Zn stress, one of the most important horticultural crops, remains largely unknown. In this study, we revealed that 24-epibrassinolide (EBR), a bioactive BR enhanced Zn tolerance in watermelon plants, which was related to the EBR-induced increase in the fresh weight, chlorophyll content, and net photosynthetic rate (Pn) and decrease in the content of hydrogen peroxide (H_2_O_2_), malondialdehyde (MDA), and Zn in watermelon leaves. Through RNA deep sequencing (RNA-seq), 350 different expressed genes (DEG) were found to be involved in the response to Zn stress after EBR treatment, including 175 up-regulated DEGs and 175 down-regulated DEGs. The up-regulated DEGs were significantly enriched in ‘phenylpropanoid biosynthesis’ pathway (map00940) using KEGG enrichment analysis. The gene expression levels of *PAL*, *4CL*, *CCR*, and *CCoAOMT*, key genes involved in phenylpropanoid pathway, were significantly induced after EBR treatment. In addition, compared with Zn stress alone, EBR treatment significantly promoted the activities of PAL, 4CL, and POD by 30.90%, 20.69%, and 47.28%, respectively, and increased the content of total phenolic compounds, total flavonoids, and lignin by 23.02%, 40.37%, and 29.26%, respectively. The present research indicates that EBR plays an active role in strengthening Zn tolerance, thus providing new insights into the mechanism of BRs enhancing heavy metal tolerance.

## Introduction

Heavy metals (HMs) pollution is a major environmental problem around the world. HMs are transported to the aboveground part through roots and accumulated in the edible part of crops, and enter human or animal bodies through the food chain, threatening human life and health^1^. Zinc (Zn), an essential biological element, participates in plant physiological metabolism as well as in the process of protein synthesis and transport^2^. However, high concentration of Zn will cause serious environmental pollution, and excessive accumulation of Zn in plants causes serious toxicity to plants^2,3^. Excessive Zn can cause the imbalance of nutrient and water absorption of plants, change the ultrastructure of chloroplasts to inhibit plant photosynthesis, destroy the membrane system and macromolecular substances of plant cells through accumulation of reactive oxygen species (ROS), and then seriously damage the growth, development, metabolism, and other plant physiological processes, causing plant death^3,4^. For example, high concentration of Zn in *M. sativa* produces a large amount of ROS, resulting in oxidative stress and cell damage, which seriously interferes *M. sativa* growth and quality^5^. Therefore, it has become a research hotspot in the field of environmental science to control Zn pollution, study the mechanism of plant tolerance to Zn and reduce the excessive absorption of Zn by plants.

Brassinolides (BRs) is a kind of natural plant hormone widely existing in plants. It has been identified as the sixth kind of plant hormone and plays a crucial role in plant growth and development, as well as biotic and abiotic stresses^6,7^. The regulation of exogenous BRs on HMs tolerance has attracted more and more attention^8^. Previous studies have shown that BRs can eliminate ROS produced by Zn stress by combining with membrane proteins, thereby alleviating the damage effect of Zn on plants. BRs can also affect the absorption of Zn by plants by stabilizing the electrical properties of cell membranes and enzyme activities, thus reducing Zn toxicity^9^. For example, Exogenous EBR spraying can improve eggplant seedlings tolerance to Zn stress through enhancement of antioxidant enzyme activity, osmotic substance accumulation, and hormone metabolism balance^10^. Exogenous EBR further increases the levels of antioxidant enzymes, thus effectively eliminating ROS, alleviating the toxicity of Zn to soybean seedlings^11^. Recently, EBR application to *M. sativa* under Zn stress could reduce Zn accumulation, promote the response of antioxidant defense system, and actively regulate the mechanism of heavy metal detoxification^5^.

Phenylpropane metabolic pathway is an important secondary metabolic pathway in plants, and its metabolites phenolics, flavonoids, and lignin are closely related to plant stress resistance^12,13^. Phenylpropane metabolism pathway plays an antioxidant role directly or indirectly in plant tolerance to heavy metal stress, and can improve plant absorption of heavy metal ions and stress tolerance^14^. In recent years, some studies have shown that BRs can improve plant stress resistance by regulating phenylpropane metabolism. Seed priming with EBR changes the growth and phenylpropanoid pathway of flax to water deficit^15^. Leaf application of EBR alleviates rice salt stress by up regulating secondary metabolites produced via the phenylalanine biosynthesis pathway^16^. EBR plays an active role in *Colletotrichum fructicola* resistance through the induction of lignin synthesis in *Camellia sinensis*^17^. Up to now, there are few reports on the research of heavy metal resistance mediated by BRs through the regulation of phenylpropane metabolism pathway, let alone Zn stress.

In the past decade, RNA-sequencing (RNA-seq) technique, a high-throughput method, has been used to investigate the global expression proﬁles^18^, which reveal the signal transduction pathways involved in Zn stress tolerance in some plants, such as wheat^19^, barley^20^, and Arabidopsis^21^. However, the specific gene expression profile of plant Zn tolerance induced by BR is still unclear. Watermelon (*Citrullus lanatus*) is one of the most important horticultural crops. However, so far, the possibility of using EBR to improve Zn tolerance of watermelon has not been studied, let alone the regulatory mechanism. Considering the increase of Zn content in horticultural soil and the stress improvement characteristics of BRs, the molecular mechanism of EBR regulating the response of watermelon to Zn stress were studied in the current study. 0.05 µM EBR could effectively alleviate Zn stress in watermelon. The gene expression pattern regulated by EBR under Zn stress was studied by RNA-seq and the different expressed genes (DEG) was significantly enriched in ‘phenylpropanoid biosynthesis. In addition, the important enzymes and secondary metabolites involved in the phenylpropanoid biosynthesis pathway have also been proved to be related to EBR application. The results of this study will provide new insights into the mechanism of BR enhancing Zn tolerance in plants.

## Materials and methods

### Plant materials

Watermelon (*C. lanatus* L.) variety 8424 was from Anhui Fengsheng Agricultural Technology Co., Ltd. Watermelon seeds were sterilized, washed, placed on filter paper, and incubated at 25°C. After germination, the seedlings were sown in pots (diameter of 8 cm), which was filled with sterilized sand and vermiculite (3:1). Growth conditions: temperature 25 ± 2°C, relative humidity 70 ± 5%, photoperiod 12/12 h, photosynthetic photon flux density 500 μmol m^−2^ s^−1^. After 10 days, plants were irrigated with 1/2 Hoagland’s nutrient solution containing Zn or not.

### Stress treatment

The Zn (ZnSO_4_·7 H_2_O) concentration was selected based on preliminary experiment, using 1.0, 2.5, 5.0, or 10.0 mM of Zn. It was found that plant growth was inhibited at 5 mM Zn but not completely inhibited (IC50) (data not shown). The EBR concentrations were selected using 0.025, 0.05, 0.10, 0.20, or 0.50 μM EBR and 0.05 μM EBR were selected.

When the plant reached the 4-leaf stage, healthy seedlings with consistent morphology were randomly divided into two groups: control and Zn treatment group. For EBR concentration screening, the following experimental design was carried out: plants were subjected to Zn and EBR-free nutrient solution (Control), or subjected to 5.0 mM Zn with different concentration of EBR (0, 0.025, 0.05, 0.10, 0.20, or 0.50 μM EBR). Seedlings were sprayed with EBR one day in advance and were watered with the nutrient solution containing Zn every 3 days. After 10 days of Zn/EBR application, the third leaves were frozen in liquid N_2_ and kept at−80°C until analysis.

### Photosynthetic pigments and net photosynthetic rate (Pn)

The 0.1 g fresh leaf samples were homogenized in 10 ml of 80% frozen acetone at 4°C at 10，000 g centrifuge for 10 min. The absorbances of chlorophyll a and b in the supernatant at 663, 645, and 480 nm were recorded.

The Li-6400 portable photosynthetic instrument (LI-COR, Lincoln, Nebraska, USA) was used to measure Pn of the third true leaf of watermelon, and the instrument automatically recorded the net photosynthetic rate. The measured light intensity is 1,000 µmol m^−2^ s^−1^, leaf temperature is 25°C, CO_2_ concentration is 400 µmol mol^−1^, and relative humidity is 75%.

### *Content measurement of Zn, H_2_O_2_*, *and MDA*

For content measurement of Zn, the dried material was weighed, ground to a powder, and digested with a 1:3 mixture of HCl:HNO3. The digests were then dissolved in ultrapure water. Then, the digested sample were analyzed using a ﬂame-atomic absorption spectrometer (AAS, PerkinElmer) and the content was expressed as mg g^−1^ dry weight. H_2_O_2_ and MDA content were measured according to the method of Wu et al.^22^.

### RNA sequencing and differentially expressed genes (DEGs)

Total RNA from watermelon leaves of Zn+EBR and Zn treatments was isolated. After the RNA sample was qualified, the enrichment of mRNA and the construction of cDNA library were carried out. After the library inspection was qualified, the RNA-seq was carried out using an Illumina HiSeq^TM^2000 platform. Three biological replicates were performed. Each sample got more than 6 Gb of data. After sequencing, the raw data was filtered to obtain high-quality clean data. The ﬁltered clean reads were aligned to the reference genome (http://www.watermelondb.cn) using HISAT2 software^23^. Differentially expressed genes (DEGs) were analyzed using the DESeq2 package with |log2 Fold change| ≥ 1 and p-value<0.05. The fold change indicates the ratio of the expression amount between two groups.

### GO and KEGG Enrichment

BLASTALL (v2.2.26) software was used to carry out annotations of GO, KEGG, COG, KOG, NR, Swissprot, Pfam databases to analyze the DEG functions. KEGG pathway enrichment was analyzed using the KEGG database (https://www.kegg.jp/kegg/)^24^. Using R (v3.6.2) to make KEGG enrichment scatter diagram for the first 20 most significant pathways. Gene ontology (GO) enrichment of DEGs was annotated into GO database, enriching and analyzing the BP, MF, and CC functions, respectively, using Fisher exact test. GO and KEGG terms with corrected p-value<0.05 were considered significantly enriched.

### Validation by RT-qPCR

Total RNA from watermelon leaves of Zn+EBR and Zn treatments was isolated and the SuperscriptIII first-strand synthesis system (Invitrogen, Shanghai, China) was used for cDNA synthesis according to the manufacturer’s protocol. Use SYBR Premium Ex Taq (Takara, Dalian, China) to conduct RT-qPCR in real-time PCR system. The selected specific primers for DEG were designed according to the Watermelon Genome Database (http://www.watermelondb.cn), as shown in Supplementary Table S1. *β-Actin* gene was used as a reference for normalizing the data.

Determination of the activities of phenylalanine ammonia-lyase (PAL), 4-coumaric ligase (4CL), and peroxidase (POD)

Plant material was homogenized with 50 mM sodium borate buffer (pH 8.0) supplying 1 mM ethylenediaminetetraacetic acid disodium salt (EDTA) and 2% polyvinyl pyrrolidone (PVP), and then immediately centrifuged at 12,000 g and 4°C for 20 min. The supernatant fraction was frozen in liquid nitrogen and placed at−80°C for determining enzyme activities of PAL, 4CL, and POD according to the method of Shao et al.^25^. Mainly, the activities of PAL, 4CL, POD and CAD were determined by ELISA kit (JiangLai, Shanghai, China) following the protocol.

### Total phenols, total flavonoids, and lignin content

According to the method of Alam et al.^26^, the phenol content was estimated by Folin – Ciocalteu reagent, and the phenol content was expressed as mg gallic acid equivalent (GAE) g^−1^ of sample. The flavonoid was determined by sodium nitrite aluminum nitrate colorimetry with sophorin as the standard sample. The flavonoid content was expressed as mg g^−1^ sample. The lignin content was determined by spectrophotometry according to the method of Xu et al.^27^. Acetyl lignin is produced after the phenol hydroxyl in lignin is acetylated. The product has a characteristic absorption peak at 280 nm. The lignin content was measured on the basis of the changing absorbance.

### Statistical analysis

Each treatment has three biological replicates. The data is the average ± SD (Standard Deviation) of the replicates displayed by the vertical error bar. One-way analysis of variance (ANOVA) and the Least Significance Difference (LSD) test (significance level is 0.05, *P* value≤0.05) were used to analyze the differences. SPSS version 20.0 (IBM, USA) was used for statistical analysis.

## Results

### Growth and Zn accumulation

**Figure 1.**
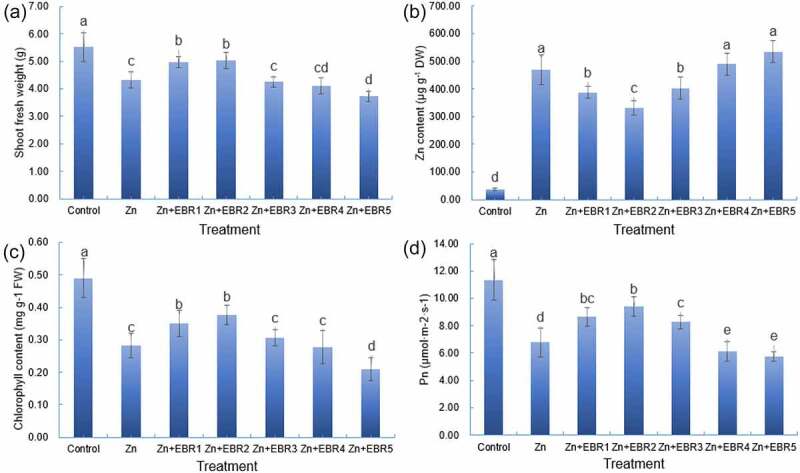
Effect of different EBR treatments on the shoot growth (a), Zn accumulation (b), the chlorophyll level (c), and Pn (d) in watermelon seedlings under Zn stress. The data is the average ± SD of three replicates displayed by the vertical error bar. Different letters in each line indicate that there is a significant difference between them (*P* ≤ 0.05) conducted by one-way analysis of variance (ANOVA) and the Least Significance Difference (LSD) test. Plants were subjected to Zn and EBR-free nutrient solution (Control), or subjected to Zn, Zn+EBR1, Zn+EBR2, Zn+EBR3, Zn+EBR4, Zn+EBR5, that means 5.0 mM Zn with different concentration of EBR (0, 0.025, 0.05, 0.10, 0.20, or 0.50 μM EBR, respectively).

To explore the effect of EBR on watermelon seedlings against Zn stress, a concentration range (0, 0.025, 0.05, 0.10, 0.20, or 0.50 μM EBR) was used. The growth of watermelon was obviously inhibited under Zn treatment alone (Figure 1a). Compared with the control, the shoot fresh weight decreased significantly. However, after pre-spraying EBR with different concentrations, the inhibition of watermelon by Zn was alleviated, which depended on the concentration effect of EBR. Compared with Zn alone, 0.025 and 0.05 significantly increased shoot fresh weight by 14.83% and 16.68%, respectively, while the growth of watermelon was inhibited by 13.82% under 0.50 μM EBR. The results showed that 0.025–0.05 EBR could alleviate Zn damage and promote seedling growth of watermelon.

EBR-induced watermelon tolerance to Zn stress is accompanied by a decrease in Zn accumulation (Figure 1b). Compared with the control, the leaves treated with Zn showed accumulation of large amount of Zn, but 0.05 μM EBR pretreatment significantly reduced Zn accumulation, presenting the best dose manner (Figure 1b).

### Chlorophyll and photosynthesis

The Chl content and Pn in watermelon seedlings under Zn treatment alone decreased significantly compared with the control (Figure 1c, d). The application of low concentration EBR, however, promoted the increase of Chl content and Pn. Compared with Zn alone, under 0.05 μM EBR treatment, Chl content, and Pn increased by 32.94% and 38.84%, respectively, while 0.50 μM EBR treatment significantly decreased Chl content and Pn. It indicates that exogenous low concentration EBR is helpful to increase the Chl content of watermelon seedlings under Zn stress, improve the net photosynthetic rate, and thus enhance the photosynthetic capacity.

### Oxidative stress

**Figure 2.**
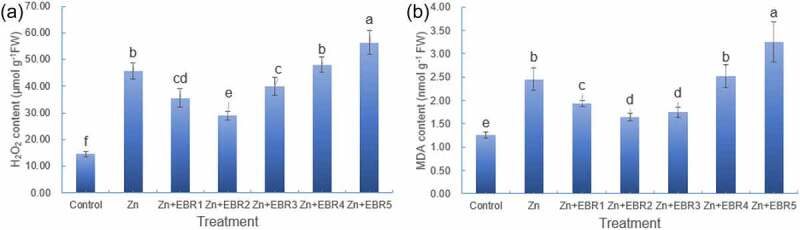
Effect of different EBR treatments on the content of H_2_O_2_ (a) and MDA (b) in watermelon seedlings under Zn stress. The data is the average ± SD of three replicates displayed by the vertical error bar. Different letters in each line indicate that there is a significant difference between them (*P* ≤ 0.05) conducted by one-way analysis of variance (ANOVA) and the Least Significance Difference (LSD) test. Plants were subjected to Zn and EBR-free nutrient solution (Control), or subjected to Zn, Zn+EBR1, Zn+EBR2, Zn+EBR3, Zn+EBR4, Zn+EBR5, that means 5.0 mM Zn with different concentration of EBR (0, 0.025, 0.05, 0.10, 0.20, or 0.50 μM EBR, respectively).

Compared with the control, Zn alone stress showed an increase in the levels of H_2_O_2_ and MDA in leaves (Figure 2). Under Zn stress, spraying exogenous EBR increased watermelon tolerance to Zn in a dose-dependent manner, and 0.05 μM was an optimum concentration for spraying watermelon seedlings to decrease oxidative stress. 0.05 μM EBR could significantly reduce the content of H_2_O_2_ and MDA in leaves by 36.57% and 32.79%, respectively, indicating that EBR might improve Zn stress tolerance by alleviating oxidative stress damage. Based on the above growth and physiological data, 0.05 μM EBR as a beneficial dose was selected for the following analysis.

### Differential expression genes (DEGs)

**Figure 3.**
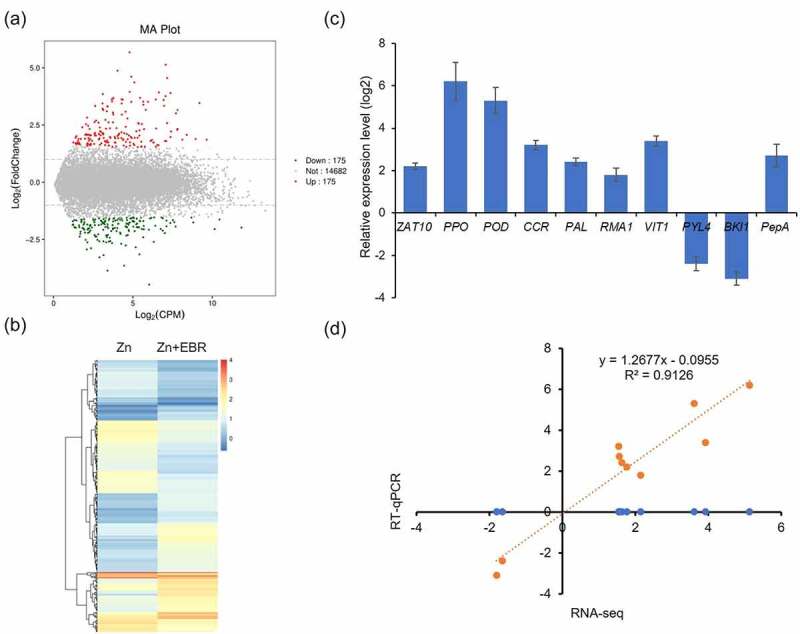
RNA-seq of differentially expressed genes (DEGs) in Zn and Zn+EBR treatment of watermelon leaves. (a) MA plot. The FDR<0.05 is used as thresholds to determine the signiﬁcance of DEGs. Red dots: up-regulated DEGs; Green dots: down-regulated DEGs; Grey dots: the transcript in the Zn+EBR library that has no significant change compared with Zn. (b) Hierarchical clustering of all the DEGs based on log10 FPKM values. (c) Verification of differentially expressed genes (DEGs) by qRT-PCR. Ten DEGs were analyzed by RT-qPCR, including *ZAT10* (ClG42_02g0071700), *RMA1* (ClG42_03g0060400), *Peroxidase 21* (ClG42_10g0201300), *CCR* (ClG42_09g0033200), *PAL* (ClG42_04g0096300), *PPO* (ClG42_03g0058500), *VIT1* (ClG42_01g0066400), *PYL4* (ClG42_04g0073000), *BKI1* (ClG42_05g0217200), and *PepA* (ClG42_02g0149200). (d) Pearson’s correlation of RNA-seq and RT-qPCR results.

Six cDNA libraries from two treatments (Zn; Zn +0.05 μM EBR) were sequenced using the Illumina HiSeq^TM^2000 platform. The raw data contained total average 7,134,478,450 baseSum. After the low-quality sequences were removed, total average 24,448,262 readSum was obtained. The average of Q20, Q30 values, and GC content was 95.9%, 91.6%, and 45.5%, respectively (Table S2). These reads were, thus, mapped to the watermelon reference genome (http://www.watermelondb.cn). The DEGs analysis was conducted with the screening threshold was: | log2FC| ≥1 and q-adjust<0.05. We identified 350 DEGs between Zn+EBR and Zn treatments (Figure 3a; Table S3) including 175 up-regulated and 175 down-regulated DEGs. Hierarchical clustering was used to observe gene expression patterns with log10 FPKMs in both groups (Figure 3b).

To verify the reliability of RNA-seq data, 10 DEGs were analyzed by RT-qPCR (Figure 3c), including *ZAT10* (ClG42_02g0071700), *RMA1* (ClG42_03g0060400), *POD* (ClG42_10g0201300), *CCR* (ClG42_09g0033200), *PAL* (ClG42_04g0096300), *PPO* (ClG42_03g0058500), *VIT1* (ClG42_01g0066400), *PYL4* (ClG42_04g0073000), *BKI1* (ClG42_05g0217200), and *PepA* (ClG42_02g0149200). The results showed that the expression level of all DEGs was consistent with the transcriptomic data and the correlation coefficient (R^2^ = 0.9126) was observed (Figure 3d), indicating the reliability of RNA-seq data.

### Functional enrichment analysis of DEGs

GO enrichment analysis was used to evaluate the function of DEG, revealing the BP, MF, and CC categories for the 350 DEGs (Table S4). A total of 175 up-regulated DEGs were signiﬁcantly enriched in four functional terms including ‘response to oxidative stress (GO:0006979)’, ‘extracellular region (GO:0005576)’, ‘cell wall (GO:0005618)’, and ‘endoplasmic reticulum lumen (GO:0005788)’. For down-regulated DEGs, metabolic process (GO:0008152) and ‘protein kinase inhibitor activity (GO:0004860)’ were signiﬁcantly enriched.

**Figure 4.**
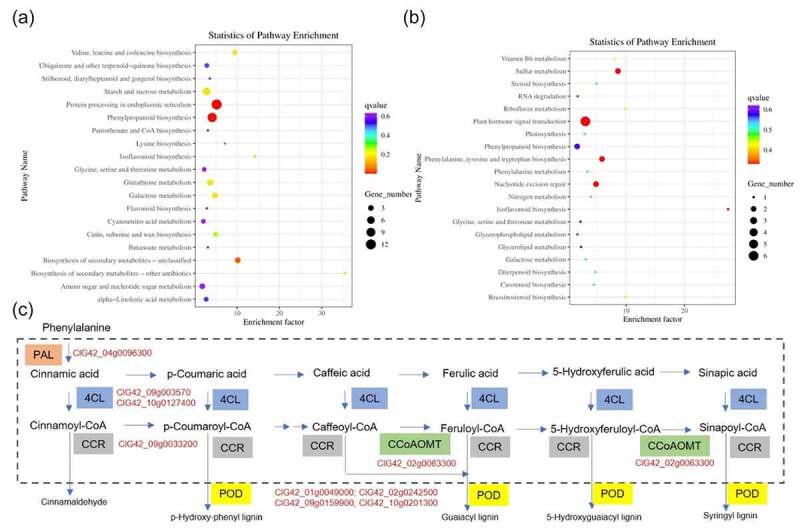
KEGG enrichment analysis of DEGs between Zn and Zn+EBR treatment of watermelon leaves. The enrichment analysis of KEGG shows the first 20 enrichment pathways of up-regulated (a) and down-regulated (b) DEGs. The left Y-axis shows the KEGG pathway. The X-axis shows the Rich factor. The meaning of the displayed q-value is represented by the color scale, where the color and size of the points represent the range of the q-value and the number of DEGs mapped to each path, respectively. (c) DEGs involved in phenylpropanoid biosynthesis (Ko00940). PAL, 4CL, CCR, COMT, and POD mediated metabolic pathway are involved in the way. PAL, phenylalanine ammonium lyase; 4CL, 4-coumarate-CoA ligase; COMT, caffeate O-methyltransferase; CCR; cinnamoyl-CoA reductase.

To check the DEG-related pathways, they were searched using the KEGG pathway database. KEGG enrichment indicated that the up-regulated DEGs were significantly associated with ‘phenylpropanoid biosynthesis (map00940)’, ‘protein processing in endoplasmic reticulum (map04141)’, and ‘biosynthesis of secondary metabolites – unclassified (map00999)’ (Figure 4a; Table S5). For down-regulated DEGs, no pathway was signiﬁcantly enriched (Figure 4b).

### Critical functional DEGs regulated by EBR under Zn stress

*DEGs related to oxidative stress*. The biological process of up-regulated DEGs was significantly enriched to ‘response to oxidative stress, such as common antioxidant enzyme genes peroxidase (ClG42_01g0049000, ClG42_10g0201300, ClG42_02g0242500, ClG42_09g0159900) and polyphenol oxidase (ClG42_03g0058500) (Table S4), suggesting that EBR-alleviated Zn stress was related to EBR-induced antioxidant protection to alleviate oxidative damage, which was consistent with the physiological data (Figures 1, 2).

*DEGs related to phenylpropanoid biosynthesis*. EBR treatment induced the changes in the expression level of some key genes under Zn stress, which were involved in phenylpropanoid biosynthesis, including phenylalanine ammonium lyase (PAL, ClG42_04g0096300), 4-coumarate-CoA ligase (4CL, ClG42_09g003570; ClG42_10g0127400), cinnamoyl-CoA reductase (CCR, ClG42_09g0033200), and caffeate O-methyltransferase (COMT, ClG42_02g0063300) (Figure 4c; Figure S1).

*DEGs related to cell wall*. The cellular component (CC) of up-regulated DEGs was significantly enriched in extracellular region and cell wall (Table S4), such as ClG42_05g0027500 encoding pectinesterase 2 and ClG42_10g0192200, ClG42_09g0179200, ClG42_08g0082000, and ClG42_07g0166400 encoding extensin-like protein enriched in external encapsulating structure and cell wall organization. Besides, ClG42_01g0049000 is a lignin-forming anionic peroxidase. PAL, 4CL, CCR, and COMT are involved in lignin biosynthetic process.

*DEGs related to protein processing in the endoplasmic reticulum (ER)*. There were 12 DEGs involved in protein processing in the ER (Figure S2; Table S5). Among them, the 11 differentially expressed HSPs and a E3 ubiquitin-protein ligase RMA1 genes were up-regulated. The DEGs were all enriched in the degradation of ubiquitin – proteasome-dependent process of ER-associated degradation (ERAD). The results indicate that ERAD is involved in EBR mediated Zn stress tolerance.

### EBR activates phenylpropanoid biosynthesis pathway

**Figure 5.**
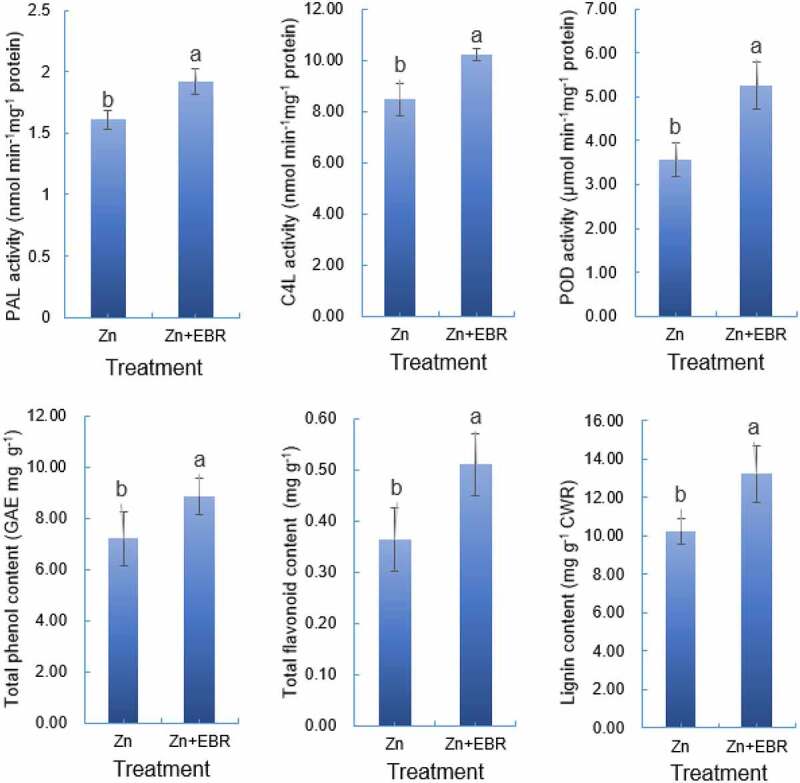
Effect of EBR on the activities of PAL, 4CL, and POD and the phenol, flavonoid and lignin in the leaves of watermelon seedlings under Zn stress. Plants were subjected to Zn and Zn+0.10 μM EBR treatment. The data is the average ± SD of three replicates displayed by the vertical error bar. Different letters in each line indicate that there is a significant difference between them (*P* ≤ 0.05) conducted by Student’s t-test.

Due to the significant enrichment in the phenylpropanoid biosynthesis pathway (Figure 4c; Figure S1), here, the activities of three key enzymes including PAL, 4CL, and POD were analyzed. The presence of EBR significantly increased the activities of PAL, 4CL, and POD of watermelon leaves by 30.90%, 20.69%, and 47.28%, respectively, under Zn stress compared to that in the absence of EBR (Figure 5). Therefore, we further measured the content of these metabolites. As shown in Figure 5, compared with Zn alone, EBR promoted the accumulation of these substances in watermelon leaves. Total phenolic compounds, total flavonoids, and lignin in Zn+EBR treated leaves were averagely 23.02%, 40.37%, and 29.26% more than those in Zn treated leaves, respectively.

## Discussion

When a large amount of Zn is enriched in the soil, it will adversely affect the growth of plants, then affect the quality and yield^1^. Here, Zn stress treatment significantly inhibited the growth of watermelon, and also significantly inhibited the accumulation of Zn, chlorophyll content, and photosynthetic rate of watermelon (Figure 1), which was consistent with the previous studies that plant growth was inhibited under Zn stress^4,5,28–30^. To reduce the serious impact of heavy metal stress (including Zn) on plants, many methods have been adopted, including foliar application of different chemicals/phytohormones^4,5,29,31–35^. BRs act as multidimensional regulators of plant responses under different environmental stresses, and epibrassinolide (EBR) is a synthetic brassinolide analog, which has been widely used in production^7^. In this study, the addition of EBR could significantly promote the increase of the biomass of watermelon seedlings under Zn stress (Figure 1), indicating that exogenous EBR treatment can alleviate the watermelon growth inhibition caused by Zn stress, and 0.05 μM EBR treatment is the best. Similar studies have been reported in other different plants^28–30^. When plants are damaged by heavy metals, on the one hand, EBR can increase the activation of H± ATPase and activate cell wall lysis enzyme, thus promoting cell growth through cell division and elongation; on the other hand, EBR can increase photosynthesis, which may be the main reason^36^.

Photosynthetic pigments are involved in the absorption, transmission, and transformation of light energy in plants, and are one of the indicators of photosynthetic capacity. When plants are in a stress environment for a long time, there will be a decrease in chlorophyll content, photosynthesis rate and other characteristics^37^. In this experiment, chlorophyll content and Pn in watermelon leaves decreased under Zn stress (Figure 1), indicating that Zn-induced decline of biomass is directly related to the decline of net photosynthetic rate of leaves. Low concentration EBR application could significantly increase the chlorophyll content and Pn in watermelon leaves, suggesting that EBR could promote the synthesis of chlorophyll or inhibit chlorophyllase activity in watermelon leaves, thus promoting the absorption and utilization of light energy in watermelon leaves^38^. Besides, EBR-induced increase in the levels of chlorophyll under stress was related to the decrease of ROS accumulation, thus reducing oxidative damage to thylakoid membrane structure and function^34^. The possible antioxidant defense mechanism for the beneficial effect of EBR application was discussed in the following sections.

Cell membrane is an important structure for plant cells to isolate protoplasts from the external environment, which can control the material exchange and signal transmission between cells and the external environments^39^. When plants are under stress, plant cells will produce excessive ROS, which will cause changes in plant membrane and damage to membrane structure. The plasma membrane and the inner membrane system of various organelles will expand or damage^39,40^. The accumulated active oxygen radicals will cause the unsaturated bonds in membrane fatty acids to be oxidized to form MDA. The level of MDA is an important indicator to reflect the strength of cells under optimal stress and the degree of plasma membrane damage^30^. Similar to other environmental stresses, excessive Zn leads to the formation of excessive ROS in plants cells resulting in plant cell oxidative damage and membrane lipid peroxidation^3,41^. This study showed that the H_2_O_2_ and MDA content of watermelon seedlings were significantly increased after being stressed by Zn (Figure 2), indicating that the cell plasma membrane had peroxidation, which disturbed the normal physiological function of the plasma membrane. However, after exogenous EBR treatment, the content of H_2_O_2_ and MDA decreased significantly, indicating that EBR had a positive role in mitigating oxidative stress damage caused by Zn stress. The biological process of up regulating DEG significantly enriched the response to oxidative stress (Table S4), which further demonstrated the mechanism of EBR mediated oxidative stress response to alleviate Zn damage.

The main way for plant systems to overcome the oxidative stress is to use primary metabolites and/or secondary metabolites^42^. On the one hand, ROS neutralization is achieved through the complex antioxidant system developed by plant cells. Stress tolerance of major metabolites is mainly through classical antioxidant enzyme system and non-enzyme system^42^. Here, under Zn stress exogenous EBR treatment could significantly increase the expression level of three peroxidase and one polyphenol oxidase genes (Table S4) and have a high activity of POD (Figure 5). Peroxidases as ‘stress enzymes’ are heme containing proteins that use H_2_O_2_ to oxidize various organic and inorganic substrates. Peroxidase activity is a sensitive indicator of heavy metal stress^43^. Polyphenol oxidase (PPO) acts as a quencher of chloroplast photooxidation. In addition, due to the vacuole position of PPO substrate, PPO may synthesize specific metabolites and react to environmental factors^44^. It is also believed that the polymerization of polyphenols by peroxidase increases after heavy metal absorption and detoxification, which is the reason for heavy metal binding in the skin gland of *Nymphaea epidermal*^45^. Alam et al.^26^ found that providing EBR to soybean plants under NaCl stress led to the enhancement of peroxidase activities coupled with the reduction in H_2_O_2_ and MDA content. These results indicated that exogenous EBR with appropriate concentration can stimulate and induce the antioxidant enzyme protection system, at least including the gene expression and activity of POD, accelerate the clearance of ROS, reduce ROS accumulation in cells, and reduce membrane lipid peroxidation damage.

On the other hand, the secondary defense also plays important roles in scavenging ROS by using a variety of secondary metabolites synthesized in plants like lignin, tannin, flavonoids, phenols, etc.^42^. These compounds are important metabolic intermediates of phenylpropane metabolic pathway. In the present study, KEGG enrichment revealed that the up-regulated DEGs were significantly associated with ‘phenylpropanoid biosynthesis (map00940)’, and ‘biosynthesis of secondary metabolites-unclassified (map00999)’ (Figure 4a; Table S5), suggesting that EBR may help to reduce the Zn toxicity in watermelon through this pathway. The genes encoding enzymes in phenylpropanoid biosynthesis including *PAL*, *4CL*, *CCR*, *CALDH*, and *CAD*, which are involved in the synthesis of flavonoids, isoflavones, and lignin, may be involved in Cd tolerance^46^. The phenylpropanoid biosynthesis pathway starts from phenylalanine, then PAL catalyzes the conversion of phenylalanine to trans-cinnamic acid. This pathway can form other phenolic compounds like flavonoids, coumarins, lignans, and lignin. They have antioxidant characteristics and can play an important role in ROS removal and the formation of metal complexes to protect plants from abiotic stress^12^. Through a series of reactions catalyzed by 4CL, C4H, HCT, and CCoAOMT, coumarinyl-CoA is converted into p-coumaroyl-CoA, caffeoyl-CoA, and feruloyl-CoA, which are the core modules of the phenylpropanoid pathway^13^. Peroxidase catalyzes the oxidation of phenylpropanoids to phenoxy, and the subsequent nonenzymatic coupling controls the pattern and extent of polymerization^47^. Under lead stress, phenylpropanoid biosynthesis is most significantly enriched in *T. orientalis* and the expression of DEGs such as *PAL*, *C4H*, *4CL* and *CCoAOM* is up-regulated^48^. *Bacillus altitudinis* WR10 regulates the phenylpropanoid biosynthesis related genes, which may improve phenolic acids accumulation, thus protecting plant cells from copper toxicity^49^. In this study, EBR treatment up-regulated the expression levels of some key genes which were involved in phenylpropanoid biosynthesis under Zn stress, including *PAL*, *4CL*, *CCR*, and *CCoAOMT* (Figure 4c; Figure S1), and the activities of three key enzymes like PAL, 4CL, and POD (Figure 5), playing important roles in lignin, flavonoids, and phenolic compounds. The changes of content of lignin, flavonoids, and phenolics are consistent with those of these genes and enzymes (Figure 5). All these results indicated that phenylpropanoid biosynthesis is an important module involved in EBR-induced Zn tolerance.

It is speculated that there are two mechanisms for phenylpropanoid pathway to participate in EBR-mediated Zn tolerance: one is that EBR further activates phenylpropyl biosynthesis pathway, leading to the generation of various phenolic compounds, which may eliminate harmful ROS; the other is to block Zn transport in plants. The rich metabolites produced by phenylpropane biosynthesis pathway coupled with high antioxidant capacity give mulberry higher salt tolerance^50^. Flavonoids are the largest class of special phenolic compounds synthesized through phenylpropane pathway, which can improve the tolerance of different plants by minimizing oxidative damage^16^. EBR supplementation further enhanced the flavonoid content in *Glycine max*^26^ and *Camellia sinensis*^51^, and *Vitis vinifera*^52^ and *Oryza sativa*^16^. Phenolic compounds are powerful heavy metal chelators. The preferential activation of genes involved in the early phenylpropanoid pathway may also be consistent with the increase of single molecule and final lignin production. EBR activates map00940-related pathways, increases lignin content in the secondary cell wall, which is closely associated with Zn absorption and transport, thus making EBR-treated plants more tolerant. At the same time, this is consistent with the enrichment of DEGs in the extracellular region and cell wall (Table S4).

## Conclusion

The present study showed that exogenous EBR enhanced Zn tolerance in watermelon seedlings with higher levels of chlorophyll and Pn and lower accumulation of H_2_O_2_ and MDA in watermelon leaves. The comparative transcriptome data and physiological analysis revealed that the increased H_2_O_2_ scavenging and activated phenylpropanoid biosynthesis pathway contributing to the mitigation of Zn toxicity by EBR. As far as we know, this is the first study to clarify the mechanism of BR reducing Zn stress through phenylpropanoid biosynthesis pathway. In conclusion, these results enhance our understanding of the mechanism of exogenous EBR enhancing Zn tolerance and the heavy metal responsive genes identified after EBR application, providing a target for future molecular breeding.^53^

## Supplementary Material

Supplemental Material

## References

[1] Arora NK, Chauhan R. Heavy metal toxicity and sustainable interventions for their decontamination. Environ Sustain. 2021;4:1–10.

[2] Broadley MR, White PJ, Hammond JP, Zelko I, Lux A. Zinc in plants. New Phytol. 2007;173:677–702. 17286818 10.1111/j.1469-8137.2007.01996.x

[3] Kaur H, Garg N. Zinc toxicity in plants: a review. Planta. 2021;253:129. 34043068 10.1007/s00425-021-03642-z

[4] Wei C, Jiao Q, Agathokleous E, Liu H, Li G, Zhang J, Fahad S, Jiang Y. Hormetic effects of zinc on growth and antioxidant defense system of wheat plants. Sci Total Environ. 2022;807:150992. 34662623 10.1016/j.scitotenv.2021.150992

[5] Ren Y, Li X, Liang J, Wang S, Wang Z, Chen H, Tang M. Brassinosteroids and gibberellic acid actively regulate the zinc detoxification mechanism of Medicago sativa L. seedlings. BMC Plant Biol. 2023;23:1–13. 36737680 10.1186/s12870-023-04091-4 PMC9898925

[6] Bajguz A, Hayat S. Effects of brassinosteroids on the plant responses to environmental stresses. Plant Physiol Bioch. 2009;47:1–8. 10.1016/j.plaphy.2008.10.002 19010688

[7] Basit F, Liu J, An J, Chen M, He C, Zhu X, Li Z, Hu J, Guan Y. Brassinosteroids as a multidimensional regulator of plant physiological and molecular responses under various environmental stresses. Environ Sci Pollut Res Int. 2021;28:44768–44779. 34235688 10.1007/s11356-021-15087-8

[8] Kour J, Kohli SK, Khanna K, Bakshi P, Sharma P, Singh AD, Sharma A. Brassinosteroid signaling, crosstalk and, physiological functions in plants under heavy metal stress. Front Plant Sci. 2021;12:608061. 33841453 10.3389/fpls.2021.608061 PMC8024700

[9] Shahzad B, Tanveer M, Che Z, Rehman A, Cheema SA, Sharma A, Zhaorong D. Role of 24-epibrassinolide (EBL) in mediating heavy metal and pesticide induced oxidative stress in plants: a review. Ecotoxicol Environ Safety. 2018;147:935–944. 29029379 10.1016/j.ecoenv.2017.09.066

[10] He J, Wang Y, Ding H, Ge CL. Epibrassinolide confers zinc stress tolerance by regulating antioxidant enzyme responses, osmolytes, and hormonal balance in *Solanum melongena* seedlings. Braz J Bot. 2016;39:295–303.

[11] dos Santos LR, da Silva BRS, Pedron T, Batista BL, Lobato AKDS. 24-epibrassinolide improves root anatomy and antioxidant enzymes in soybean plants subjected to zinc stress. J Soil Sci Plant Nutr. 2020;20:105–124.

[12] Sharma A, Shahzad B, Rehman A, Bhardwaj R, Landi M, Zheng B. Response of phenylpropanoid pathway and the role of polyphenols in plants under abiotic stress. Molecules. 2019;24:2452. 31277395 10.3390/molecules24132452 PMC6651195

[13] Vogt T. Phenylpropanoid biosynthesis. Mol Plant. 2010;3:2–20. 20035037 10.1093/mp/ssp106

[14] Berni R, Luyckx M, Xu X, Legay S, Sergeant K, Hausman JF, Guerriero G. Reactive oxygen species and heavy metal stress in plants: impact on the cell wall and secondary metabolism. Environ Exp Bot. 2019;161:98–106.

[15] Aghaee P, Rahmani F. Seed priming with 24-epibrassinolide alters growth and phenylpropanoid pathway in flax in response to water deficit. J Agr Sci Tech. 2020;22:1039–1052.

[16] Shahzad R, Harlina PW, Ewas M, Zhenyuan P, Nie X, Gallego PP, Jia H. Foliar applied 24-epibrassinolide alleviates salt stress in rice (*Oryza sativa* L.) by suppression of ABA levels and upregulation of secondary metabolites. J Plant Inter. 2021;16:533–549.

[17] Zhang L, Zhang Z, Ahammed GJ, Wang X, Fang H, Yan P, Li X. 24-Epibrassinolide enhances resistance against *Colletotrichum fructicola* by promoting lignin biosynthesis in *Camellia sinensis*. J Plant Growth Regul 2022. doi:10.1007/s00344-022-10640-2.

[18] Everaert C, Luypaert M, Maag JLV, Cheng QX, Dinger ME, Hellemans J, Mestdagh P. Benchmarking of RNA-sequencing analysis workflows using whole-transcriptome RT-qPCR expression data. Sci Rep. 2017;7:1559. 28484260 10.1038/s41598-017-01617-3 PMC5431503

[19] Wang Y, Wang X, Wang C, Peng F, Wang R, Xiao X, Zhou Y. Transcriptomic profiles reveal the interactions of Cd/Zn in dwarf polish wheat (*Triticum polonicum* L.) roots. Front Physiol. 2017;8:168. 28386232 10.3389/fphys.2017.00168 PMC5362637

[20] Kintlová M, Blavet N, Cegan R, Hobza R. Transcriptome of barley under three different heavy metal stress reaction. Genom Data. 2017;13:15–17. 28626638 10.1016/j.gdata.2017.05.016 PMC5460742

[21] Wan J, Wang R, Wang R, Ju Q, Wang Y, Xu J. Comparative physiological and transcriptomic analyses reveal the toxic effects of ZnO nanoparticles on plant growth. Environ Sci Technol. 2019;53:4235–4244. 30871319 10.1021/acs.est.8b06641

[22] Wu QS, Xia RX, Zou YN. Reactive oxygen metabolism in mycorrhizal and non-mycorrhizal citrus (*Poncirus trifoliata*) seedlings subjected to water stress. J Plant Physiol. 2006;163:1101–1110. 17032615 10.1016/j.jplph.2005.09.001

[23] Kim D, Paggi JM, Park C, Bennett C, Salzberg SL. Graph-based genome alignment and genotyping with HISAT2 and HISAT-genotype. Nat Biotechnol. 2019;37:907–915. 31375807 10.1038/s41587-019-0201-4 PMC7605509

[24] Xie C, Mao X, Huang J, Ding Y, Wu J, Dong S, Kong L, Gao G, Li CY, Wei L. KOBAS 2.0: a web server for annotation and identiﬁcation of enriched pathways and diseases. Nucleic Acids Res. 2011;39:316–322. 10.1093/nar/gkr483 PMC3125809 21715386

[25] Shao R, Zhang J, Shi W, Wang Y, Tang Y, Liu Z, Sun W, Wang H, Guo J, Meng Y. Mercury stress tolerance in wheat and maize is achieved by lignin accumulation controlled by nitric oxide. Environ Pollut. 2022;307:119488. 35597486 10.1016/j.envpol.2022.119488

[26] Alam P, Albalawi TH, Altalayan FH, Bakht MA, Ahanger MA, Raja V, Ashraf M, Ahmad P. 24-epibrassinolide (EBR) confers tolerance against NaCl stress in soybean plants by up-regulating antioxidant system, ascorbate-glutathione cycle, and glyoxalase system. Biomol. 2019;9:640. 10.3390/biom9110640 PMC6920941 31652728

[27] Xu T, Zhang S, Du K, Yang J, Kang X. Insights into the molecular regulation of lignin content in Triploid poplar Leaves. Int J Mol Sci. 2022;23:4603. 35562994 10.3390/ijms23094603 PMC9099847

[28] Ramakrishna B, Rao SSR. Foliar application of brassinosteroids alleviates adverse effects of zinc toxicity in radish (*Raphanus sativus* L.) plants. Protoplasma. 2015;252:665–677. 25308099 10.1007/s00709-014-0714-0

[29] Wu XX, Chen JL, Xu S, Zhu ZW, Zha DS. Exogenous 24-epibrassinolide alleviates zinc-induced toxicity in eggplant (*Solanum melongena* L.) seedlings by regulating the glutathione-ascorbate-dependent detoxification pathway. J Hortic Sci Biotech. 2016;91:412–420.

[30] Zhang Y, Liao H. Epibrassinolide improves the growth performance of *Sedum lineare* upon Zn stress through boosting antioxidative capacities. PLoS ONE. 2021;16:e0257172. 34492083 10.1371/journal.pone.0257172 PMC8423314

[31] Ahanger MA, Mir RA, Alyemeni MN, Ahmad P. Combined effects of brassinosteroid and kinetin mitigates salinity stress in tomato through the modulation of antioxidant and osmolyte metabolism. Plant Physiol Bioch. 2020;147:31–42. 10.1016/j.plaphy.2019.12.007 31838316

[32] Hasan SA, Hayat S, Ali B, Ahmad A. 28-Homobrassinolide protects chickpea (*Cicer arietinum*) from cadmium toxicity by stimulating antioxidants. Environ Pollut. 2008;51:60–66. 10.1016/j.envpol.2007.03.006 17481788

[33] Jan S, Alyemeni MN, Wijaya L, Alam P, Siddique KH, Ahmad P. Interactive effect of 24-epibrassinolide and silicon alleviates cadmium stress via the modulation of antioxidant defense and glyoxalase systems and macronutrient content in Pisum sativum L. seedlings. BMC Plant Biol. 2018;18:1–18. 30012086 10.1186/s12870-018-1359-5 PMC6048797

[34] Maia CF, Pereira YC, da Silva BRS, Batista BL, Lobato AKDS. Exogenously applied 24-epibrassinolide favours stomatal performance, ROS detoxification and nutritional balance, alleviating oxidative damage against the photosynthetic apparatus in tomato leaves under nickel stress. J Plant Growth Regul. 2022. doi:10.1007/s00344-022-10693-3.

[35] SurgunAcar Y, ZemheriNavruz F. Exogenous application of 24epibrassinolide improves manganese tolerance in *Arabidopsis thaliana* L. via the modulation of antioxidant system. J Plant Growth Regul. 2022;41:546–557.

[36] Alam P, Balawi TA, Ashraf M, Ahmad P. 24-Epibrassinolide (EBR) reduces oxidative stress damage induced by cadmium toxicity by restricting cd uptake and modulating some key antioxidant enzymes in maize plants. Pak J Bot. 2021;53:59–66.

[37] Ramakrishna B, Rao SSR. 24-Epibrassinolide alleviated zincinduced oxidative stress in radish (*Raphanus sativus* L.) seedlings by enhancing antioxidative system. Plant Growth Regul. 2012;68:249–259.

[38] Hayat S, Yadav S, Wani AS, Irfan M, Ahmad A. Comparative effect of 28-homobrassinolide and 24-epibrassinolide on the growth, carbonic anhydrase activity and photosynthetic efficiency of *Lycopersicon esculentum*. Photosynthetica. 2011;49:397–404.

[39] Mittler R, Zandalinas SI, Fichman Y, Van Breusegem F. Reactive oxygen species signalling in plant stress responses. Nat Rev Mol Cell Bio. 2022;23:663–679. 35760900 10.1038/s41580-022-00499-2

[40] Farooq MA, Niazi AK, Akhtar J, Farooq M, Souri Z, Karimi N, Rengel Z. Acquiring control: the evolution of ROS-Induced oxidative stress and redox signaling pathways in plant stress responses. Plant Physiol Bioch. 2019;141:353–369. 10.1016/j.plaphy.2019.04.039 31207496

[41] Jin XF, Yang XE, Islam E, Liu D, Mahmood Q, Li H, Li J. Ultrastructural changes, zinc hyperaccumulation and its relation with antioxidants in two ecotypes of *Sedum alfredii* Hance. Plant Physiol Bioch. 2008;46:997–1006. 10.1016/j.plaphy.2008.06.012 18693116

[42] Yaqoob U, Jan N, Raman PV, Siddique KH, John R. Crosstalk between brassinosteroid signaling, ROS signaling and phenylpropanoid pathway during abiotic stress in plants: does it exist? Plant Stress . 2022;4:100075.

[43] Bhaduri AM, Fulekar MH. Antioxidant enzyme responses of plants to heavy metal stress. null. 2012;11:55–69.

[44] Chen S, Wang Q, Lu H, Li J, Yang D, Liu J, Yan C. Phenolic metabolism and related heavy metal tolerance mechanism in *Kandelia obovata* under Cd and Zn stress. Ecotox Environ Safe. 2019;169:134–143. 10.1016/j.ecoenv.2018.11.004 30445244

[45] Lavid N, Schwartz A, Yarden O, Tel-Or E. The involvement of polyphenols and peroxidase activities in heavy-metal accumulation by epidermal glands of the waterlily (Nymphaeaceae). Planta. 2001;212:323–331. 11289596 10.1007/s004250000400

[46] Qiao K, Liang S, Wang F, Wang H, Hu Z, Chai T. Effects of cadmium toxicity on diploid wheat (*Triticum urartu*) and the molecular mechanism of the cadmium response. J Hazard Mater. 2019;374:1–10. 30974226 10.1016/j.jhazmat.2019.04.018

[47] Russell WR, Burkitt MJ, Scobbie L, Chesson A. EPR investigation into the effects of substrate structure on peroxidase-catalyzed phenylpropanoid oxidation. Biomacromolecules. 2006;7:268–273. 16398524 10.1021/bm050636o

[48] Xu X, Chen Q, Mo S, Qian Y, Wu X, Jin Y, Ding H. Transcriptome-wide modulation combined with morpho-physiological analyses of *Typha orientalis* roots in response to lead challenge. J Hazard Mater. 2020;384:121405. 31629596 10.1016/j.jhazmat.2019.121405

[49] Yue Z, Chen Y, Chen C, Ma K, Tian E, Wang Y, Liu H, Sun Z. Endophytic *Bacillus altitudinis* WR10 alleviates Cu toxicity in wheat by augmenting reactive oxygen species scavenging and phenylpropanoid biosynthesis. J Hazard Mater. 2021;405:124272. 33097348 10.1016/j.jhazmat.2020.124272

[50] Gan T, Lin Z, Bao L, Hui T, Cui X, Huang Y, Wang H, Su C, Jiao F, Zhang M, et al. Comparative proteomic analysis of tolerant and sensitive varieties reveals that phenylpropanoid biosynthesis contributes to salt tolerance in Mulberry. Int J Mol Sci. 2021;22:9402. 34502318 10.3390/ijms22179402 PMC8431035

[51] Li X, Ahammed GJ, Li ZX, Zhang L, Wei JP, Shen C, Yan P, Zhang LP, Han WY. Brassinosteroids improve quality of summer tea (*Camellia sinensis* L.) by balancing biosynthesis of polyphenols and amino acids. Front Plant Sci. 2016;7:1304. 10.3389/fpls.2016.01304 PMC5003824 27625668

[52] Xi ZM, Zhang ZW, Huo SS, Luan LY, Gao X, Ma LN, Fang YL. Regulating the secondary metabolism in grape berry using exogenous 24-epibrassinolide for enhanced phenolics content and antioxidant capacity. Food Chem. 2013;141:3056–3065. 23871059 10.1016/j.foodchem.2013.05.137

[53] Arenas-Lago D, Carvalho LC, Santos ES, Abreu MM. The physiological mechanisms underlying the ability of *Cistus monspeliensis* L. from São Domingos mine to withstand high Zn concentrations in soils. Ecotox Environ Safe. 2016;129:219–227. 10.1016/j.ecoenv.2016.03.041 27054705

