# Supplementary figures and images for: Exogenous Brassinosteroid Enhances Zinc tolerance by activating the Phenylpropanoid Biosynthesis pathway in Citrullus lanatus L
Source: Plant Signal Behav. 2023 Apr 21;18(1):2186640. doi: 10.1080/15592324.2023.2186640 (PMC10124981; doi:10.1080/15592324.2023.2186640)

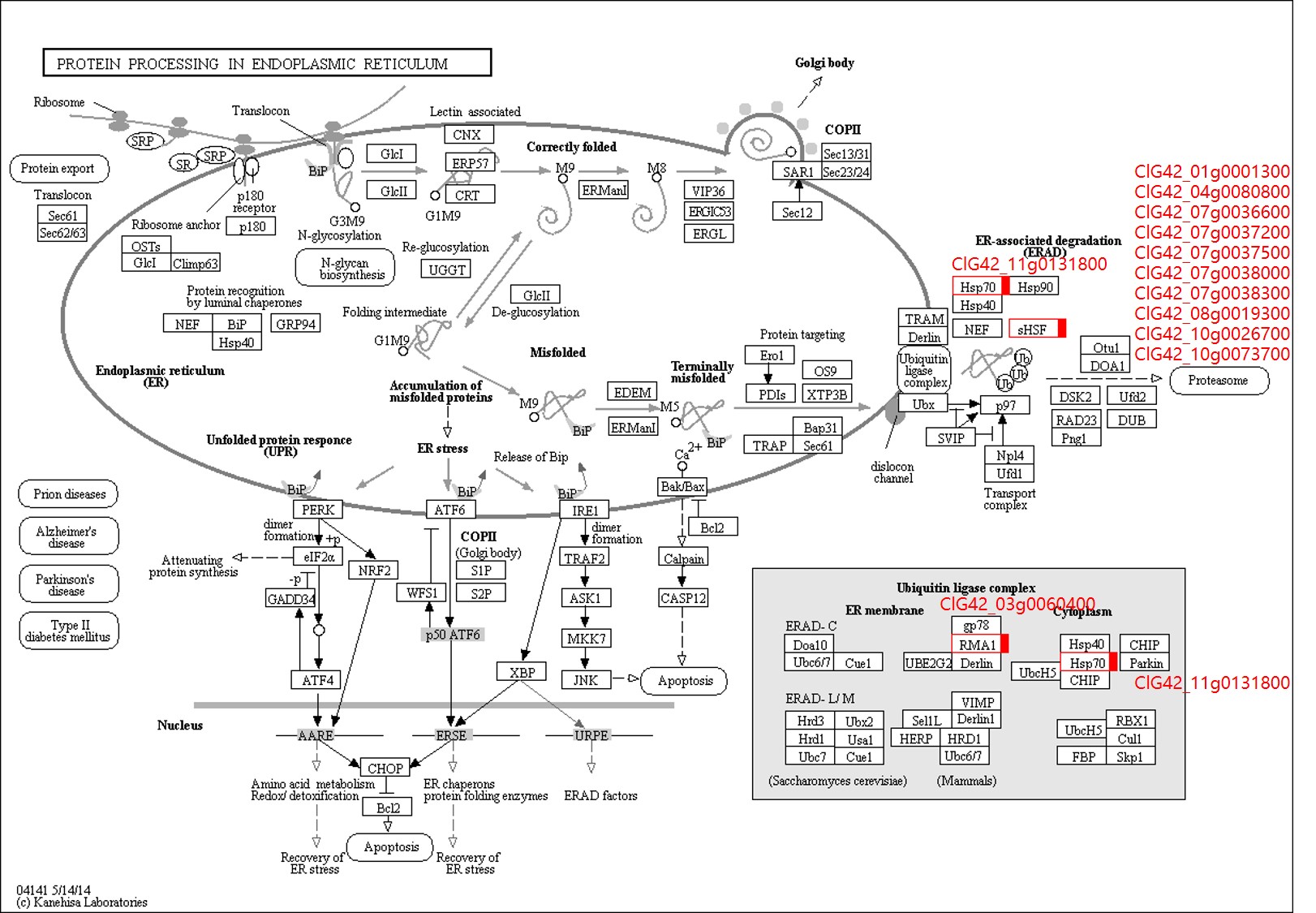

Supplement: Supplemental Material [file KPSB_A_2186640_SM1421.zip › Figure S2 DEGs involved in protein processing in the endoplasmic reticulum (map04141).jpg]

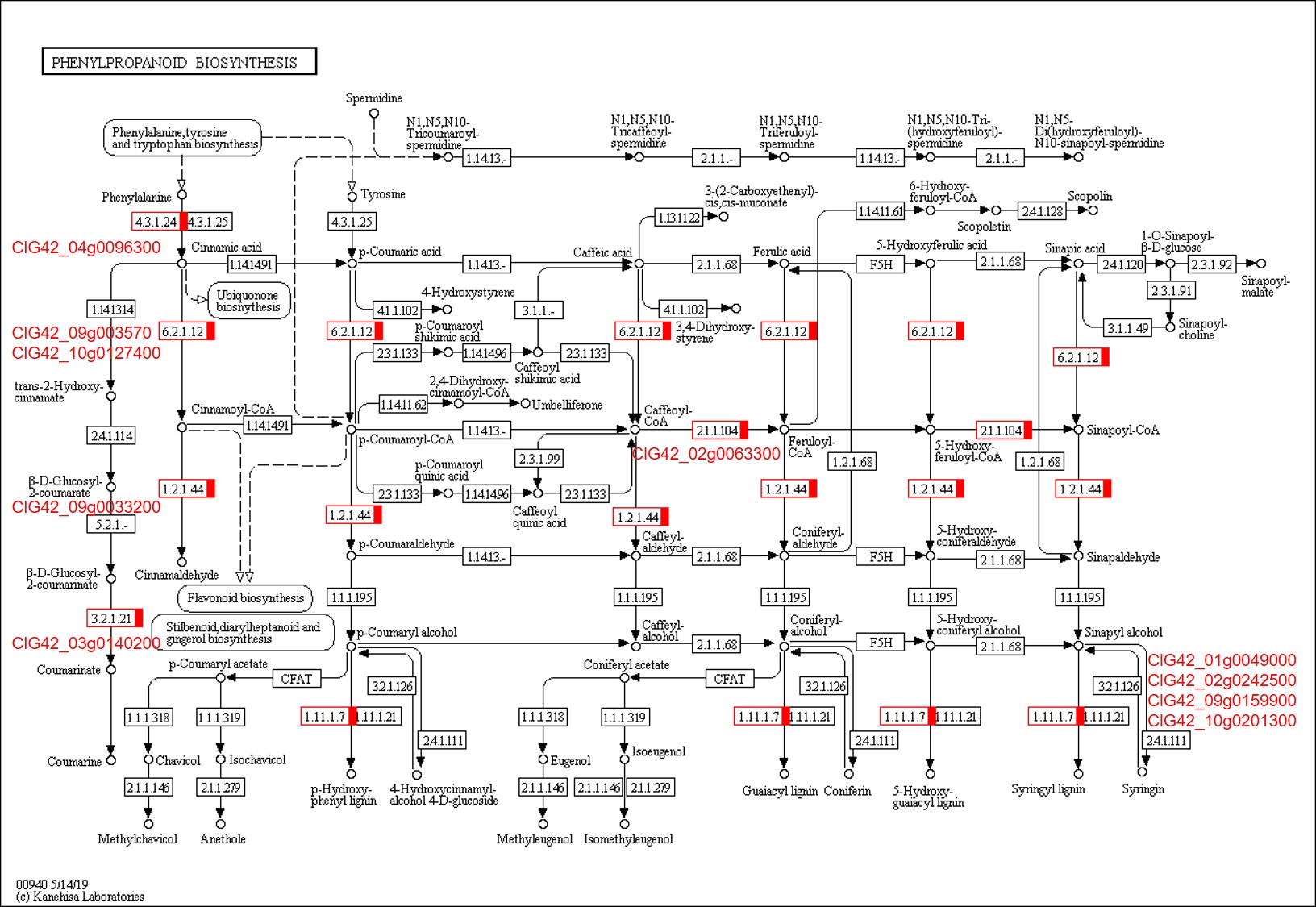

Supplement: Supplemental Material [file KPSB_A_2186640_SM1421.zip › Figure S1 DEGs involved in phenylpropanoid phenylpropanoid biosynthesis (Ko00940). Red represents up-regulation expression.jpg]
